# A Round-Efficient Authenticated Key Agreement Scheme Based on Extended Chaotic Maps for Group Cloud Meeting

**DOI:** 10.3390/s17122793

**Published:** 2017-12-03

**Authors:** Tsung-Hung Lin, Chen-Kun Tsung, Tian-Fu Lee, Zeng-Bo Wang

**Affiliations:** 1 Department of Computer Science and Information Engineering, National Chin-Yi University of Technology, No.57, Sec. 2, Zhongshan Rd., Taiping District, Taichung 41170, Taiwan; duke@ncut.edu.tw (T.-H.L.); scottzxk20@gmail.com (Z.-B.W.); 2 Department of Medical Informatics, Tzu Chi University, No.701, Sec. 3, Zhongyang Rd., Hualien 97004, Taiwan; jackytflee@mail.tcu.edu.tw

**Keywords:** cloud meeting, group authenticated, key agreement, extended chaotic maps

## Abstract

The security is a critical issue for business purposes. For example, the cloud meeting must consider strong security to maintain the communication privacy. Considering the scenario with cloud meeting, we apply extended chaotic map to present passwordless group authentication key agreement, termed as Passwordless Group Authentication Key Agreement (PL-GAKA). PL-GAKA improves the computation efficiency for the simple group password-based authenticated key agreement (SGPAKE) proposed by Lee et al. in terms of computing the session key. Since the extended chaotic map has equivalent security level to the Diffie–Hellman key exchange scheme applied by SGPAKE, the security of PL-GAKA is not sacrificed when improving the computation efficiency. Moreover, PL-GAKA is a passwordless scheme, so the password maintenance is not necessary. Short-term authentication is considered, hence the communication security is stronger than other protocols by dynamically generating session key in each cloud meeting. In our analysis, we first prove that each meeting member can get the correct information during the meeting. We analyze common security issues for the proposed PL-GAKA in terms of session key security, mutual authentication, perfect forward security, and data integrity. Moreover, we also demonstrate that communicating in PL-GAKA is secure when suffering replay attacks, impersonation attacks, privileged insider attacks, and stolen-verifier attacks. Eventually, an overall comparison is given to show the performance between PL-GAKA, SGPAKE and related solutions.

## 1. Introduction

Communicating over the Internet is a convenient application as the development of the Internet becomes popular. People can communicate with each other via cloud meeting is a common application. A lot of companies deploy cloud meeting equipment to realize a remote discussion. Some special industries also take into account the cloud meeting, but they focus on the information security. For example, personalized information must be under controlled in medical conferences, and business confidentiality can not be tapped in cloud meeting.

The cloud meeting has following properties:

1. Known members: The meeting members are known before meeting. Therefore, the organizer has a participant list in advance.
2. Difficult preset: Even if the organizer has a participant list, generating the meeting setting, e.g., passwords or meeting tokens, in advance is inappropriate. Dynamically generating meeting setting is the optimal solution [1,2,3,4,5,6,7] for the security consideration.
3. Over the Internet: This is the core requirement for realizing a cloud meeting.
4. Multi-member communication: The communication within a pair of members is tractable. When the number of participants increases, to ensure that each member can identify each other is difficult.

**Figure 1:**
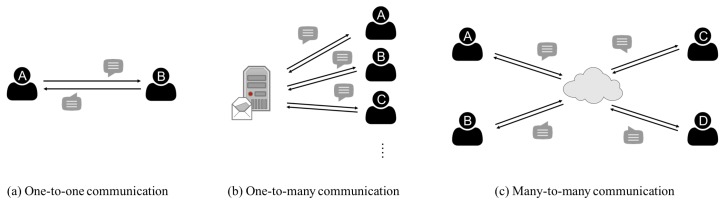
Three types of cloud meeting.

For the fourth property, the cloud meeting can be classified into three categories, and they are one-to-one, one-to-many, and many-to-many models as shown in Figure 1. The most popular application is one-to-many model. For example, the user uses a password to log in to a web service. In this model, participants have a security communication based on a centralized server [8]. The many-to-many model is similar to the one-to-many model, but the many-to-many model is decentralized [9].

Both one-to-many and many-to-many models are popular in real world cloud meeting. For example, building a safety communication tunnel to avoid information loss is a possible solution [8]. As shown in case of Figure 1b, the server provides a safety communication tunnel for all connected members. The major advantage of the one-to-many model is the convenience. Although the many-to-many model does not suffer the attacks from hackers due to the decentralization, each member must have higher security equipment in the many-to-many model than in the one-to-many model. Therefore, we focus on the one-to-many model and propose a lightweight solution with security communication.

Before entities send messages with each other, they have to build up a secure communication. In the current secure communication technologies including Internet Protocol Security (IPSec) and https require a communication setup process with two steps: session key generation and message encryption/decryption. The goal of session key generation is to compute a session key for all communication members. Since the message that required by computing a session key is sent over the Internet, hiding the information applied to generate a session key is the major challenge. After all members have the same session key, they can use the session key to encrypt or decrypt messages in the second step. In this paper, we focus on the first step to design an efficient session key agreement scheme under the scenario drawn in case of Figure 1b.

Group authentication key agreement scheme is a possible solution in security cloud meeting. Each participant generates a session key to encrypt information, and it only can be used during this cloud meeting. Even if encrypted messages sent over the Internet are taken by man in the middle, they do not have enough information to get the original message. Diffie–Hellman key exchange is an appropriate technique to develop the group authentication key agreement scheme [8]. It guarantees high security for information exchange in a limited time period. A cloud meeting takes a few hours rather than several years, so Diffie–Hellman key exchange is secure for a cloud meeting.

However, Diffie–Hellman key exchange applies modular exponentiation to compute single-use session key, so it requires a lot of computation cost before the information exchange. In the cloud meeting, the schedules of many people may be rush, so they need an efficient solution for minimizing the setup time.

Another efficient key agreement protocol is extended chaotic map-based approaches [8,10]. These kind of schemes apply Chebyshev polynomials to provide the property, which is equivalent to the semigroup property of chaotic map [10,11,12,13,14]. The details are shown in Section 2 Preliminaries. Therefore, extended chaotic map-based approaches are efficient in computing session keys [15]. However, there is no group authentication key agreement scheme that applies the extended chaotic map in the one-to-many model [10].

There are some key agreement protocols that can be applied in case of Figure 1a. For example, Abdalla and Pointcheval provide a password-based approach for a pair of users [15]. Dutta and Barua extend the results of Abdalla and Pointcheval from one-to-one communication to the many-to-many model, and the shared password has been enhanced [16]. Kim et al. focus on the members join/leave a group without the assistance from a central server [3]. Boyd and Nieto address the efficiency of the key agreement protocol in terms of the number of rounds to generate a session key, and the proposed solution can be done in one round [17]. However, the solution still needs to be improved for the forward security issue.

For the group authentication, Lee et al. present a simple group password-based authenticated key agreement (SGPAKE) [8]. SGPAKE considers modular exponentiation, but the cost of generating session keys is not acceptable in cloud meeting. Therefore, we apply the extended chaotic map to propose the passwordless group authentication key agreement, termed by PL-GAKA. PL-GAKA is an extended chaotic map-based approach, so it improves the computation efficiency of SGPAKE. Since PL-GAKA is passwordless, meeting members do not need other password maintenance.

In our analysis, we first prove that each member can compute correct session key and they have security communication. Then, we refer to [8,18,19,20,21,22] to measure the security of PL-GAKA in terms of session key security, mutual authentication, perfect forward security, data integrity, and man-in-the-middle attack. Moreover, we also demonstrate that the proposed solution is safe when suffering replay attacks, impersonation attacks, privileged insider attacks, and stolen-verifier attacks.

The structure of this paper is as follows: the background knowledge is present in Section 2. The proposed PL-GAKA is illustrated in Section 3. In Section 4, we analyze the correctness, security, and the overall comparison. The conclusion and future works are illustrated in Section 5.

## 2. Preliminaries

In this section, we will show that the security of Diffie–Hellman key exchange, and how the chaotic map-based approaches can reduce the computation cost without sacrificing the security of key agreement. In the following context, we first give an example to show the way of computing a session key over the Internet. Then, we introduce the Diffie-Hellman problem, which is the major property to guarantee the communication security. Eventually, we show an alternative technique named by the chaotic map to reduce the computation cost.

Diffie–Hellman key exchange is a famous scheme in terms of security communications. Considering the following scenario of generating a session key before starting a safety communication: Alice and Bob would like to create a security communication within *G* rounds. Firstly, Alice selects a big prime *p* and a primitive root *g*. Then, Alice generates a secrete value *a* for this communication with Bob:

Step 1 Alice obtains the message $A=g^{a}\;\mathrm{mod}\;p$ and sends g,p,A to Bob over Internet.
Step 2 Bob also computes a secret value *b* for the communication with Alice. Bob computes the message $B=g^{b}\;\mathrm{mod}\;p$ and sends *B* to Alice. Moreover, Bob uses g,p,A and *b* to compute the session key $K=A^{b}\;\mathrm{mod}\;p=g^{ab}\;\mathrm{mod}\;p$.
Step 3 Alice can compute the session key $K=B^{a}\;\mathrm{mod}\;p=g^{ba}\;\mathrm{mod}\;p$ from *B*. Then, both Alice and Bob have the same session key and they can start to communicate with each other.

In Step 3, Alice and Bob get the session key *K*, and then they can communicate with each other via encrypting/decrypting messages by *K*.

During the steps above, Alice and Bob focus on computing *K* in an open environment. Only Alice and Bob can derive correct *K* even if eavesdroppers capture the messages sent from Alice or Bob. The core idea of the safety in terms of generating *K* is the Diffie–Hellman problem and that is shown in the following definition.

**Definition** **1.** Diffie–Hellman problem [23]: Given appropriate settings of G and g, eavesdroppers obtain $g^{ab}$ by solving the Diffie–Hellman problem.

Solving Difie-Hellman problem is hard [23,24], and this is the reason that Diffie–Hellman key exchange provides high security. However, Diffie–Hellman key exchange requires heavy computation cost due to the modular exponentiation consideration. Designing a key ageerment approach with lower computation cost is a research direction.

Since Alice computes $K=B^{a}\;\mathrm{mod}\;p=g^{ba}\;\mathrm{mod}\;p$ and Bob computes $K=A^{b}\;\mathrm{mod}\;p=g^{ab}\;\mathrm{mod}\;p$, they derive the same *K*. Therefore, Alice and Bob can generate the session key via Internet. Chebyshev polynomials have similar properties as shown in the following definition.

**Definition** **2.** Semigroup property [25]: We have $T_{r}\left(T_{s}\left(x\right)\right)=T_{rs}\left(x\right)$ for different r and s, where ∀−1≤x≤1.

The core idea of semigroup is similar to $g^{ab}$ in the Diffie–Hellman problem. Semigroup implies that there is not a specific order for *r* and *s*. This property comes from Chebyshev polynomials, which is defined as $T_{n+1}\left(x\right)=2xT_{n}\left(x\right)-T_{n-1}\left(x\right)$, where $T_{0}\left(x\right)=1$, $T_{1}\left(x\right)=x$, $n\in Z^{+}$, and x∈R.

However, −1≤x≤1 is not enough provide high security in terms of the diversity of *x*, and Zhang extends the mapping range from [−1,1] to (−∞,∞) [10]. The Extended Chebyshev polynomials are shown in Definition 3. The security can be improved dramatically. In other words, the scheme with semigroup property has similar security to that of the Diffie–Hellman key exchange.

**Definition** **3.** Extended Chebyshev polynomials: Given x∈R, we have $T_{r}\left(T_{s}\left(x\right)\right)\;\mathrm{mod}\;p=T_{sr}\left(x\right)\;\mathrm{mod}\;p=T_{s}\left(T_{r}\left(x\right)\right)\;\mathrm{mod}\;p$ for different r and s.

In other words, we can apply chaotic map functions to design a key agreement approach with lower computation costs than that required by Diffie–Hellman key exchange protocols. The chaotic map-based key agreement approaches have similar security to that of the Diffie–Hellman problem.

## 3. Proposed Solution

SGPAKE has three processes including registration, authentication, and password modification. PL-GAKA is a passwordless scheme, so password modification is not necessary. The processes of registration and authentication are illustrated in the following subsections. Moreover, the symbol system applied in this paper is shown in Table 1.

### 3.1. Registration

The purpose of registration is to construct a list of potential meeting members for GWN. Each meeting member $U_{i}$ provides the identity $UID_{i}$ to GWN. GWN uses $UID_{i}$ to generate the encrypted shared secret information $K_{GS_{i}}$, and then $U_{i}$ are available to join a cloud meeting.

**Figure 2:**
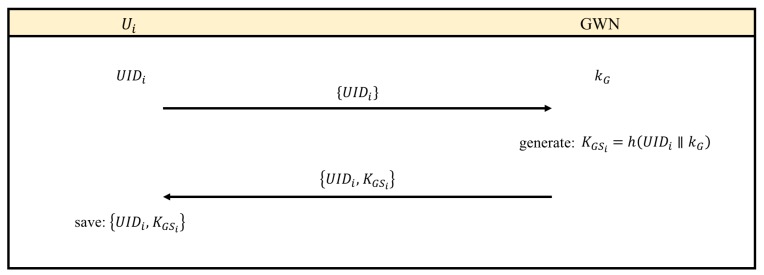
The registration process.

The major consideration is the security, and we have the following issues. The first issue is how GWN confirms $U_{i}$, and the second one is how to ensure the safety of the entire process. Since PL-GAKA is passwordless, $UID_{i}$ is important information for verifying $U_{i}$. The whole registration can be completed in an offline and face-to-face process, and the secure solution can be applied to determine the user characteristics, e.g., smart card [18]. We focus on providing the communication security during the cloud meeting, and meeting members can be pre-defined before meeting. Therefore, the offline registration process is available for cloud meeting to ensure each member is verified. The registration processes are illustrated in Figure 2, and details are listed as follows:

Step 1 The user $U_{i}$ registers his/her identity $UID_{i}$ in GWN.
Step 2 GWN uses the private key $k_{G}$ to compute $K_{GS_{i}}=h\left(UID_{i}∥k_{G}\right)$ and then sends $K_{GS_{i}}$ to $U_{i}$ via the secure channel.
Step 3 $U_{i}$ saves $K_{GS_{i}}$ for further authentications.

### 3.2. Authentication

The communication security depends on the stable member. All members must know each other. When a member joins the meeting, the authentication process is launched to ensure that all members know each other including GWN.

The authentication process spreads four messages. In the beginning, each $U_{i}$ sends the encrypted identity message $M_{1}$ to GWN. GWN verifies $M_{1}$ and sends the message $M_{2}$ including the list of meeting members and the encrypted server information back. After receiving $M_{2}$, $U_{i}$ broadcasts $M_{3}$ including the information required by cross authentication. Then, $U_{i}$ generates and broadcasts authentication information $M_{4}$. Eventually, each member authenticates each other and computes the session key for the encryption in the following meeting. We consider the timestamp in each message to guarantee that the process sequence can be tracked. Thus, when receiving a message, verifying the timestamp is the first task.

**Figure 3:**
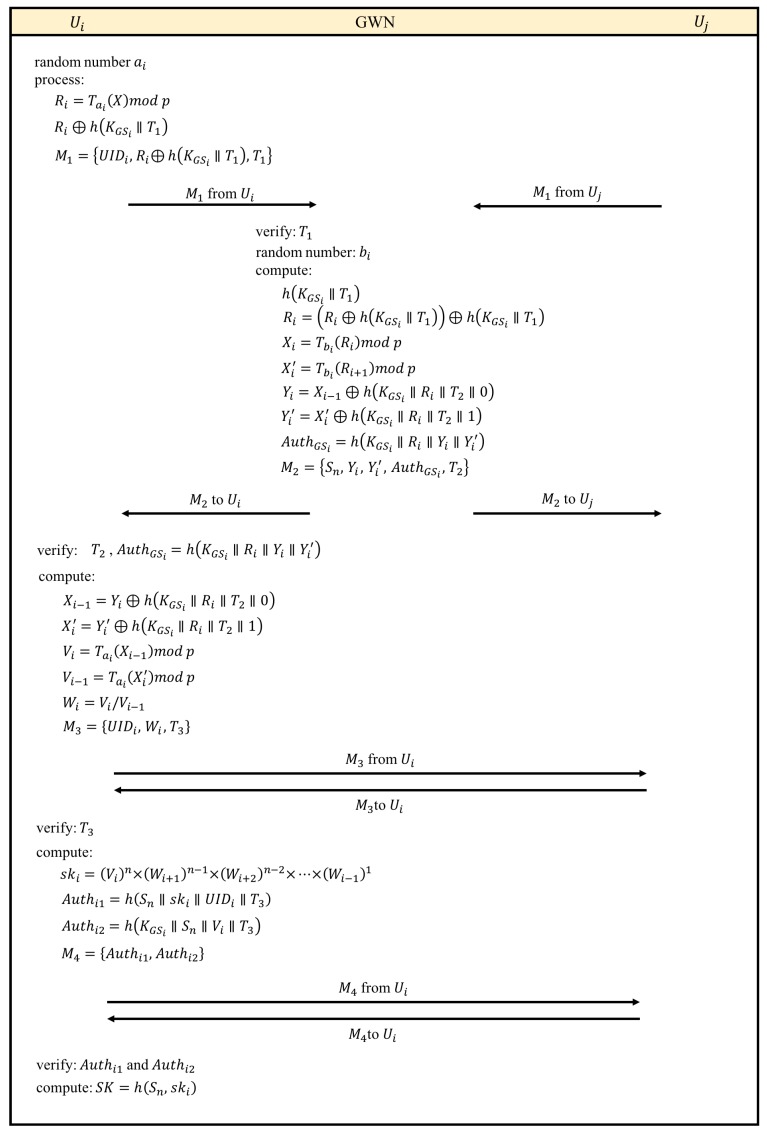
The authentication process.

Consider *n* registered members who would like to participate in a cloud meeting. The proposed authentication process is illustrated in Figure 3, and the details are shown as follows:

Step 1 Each user $U_{i}$ generates a random number $a_{i}$ and computes $R_{i}=T_{a_{i}}\left(X\right)\;\mathrm{mod}\;p$. After considering the timestamp $T_{1}$, we have $R_{i}⊕h\left(K_{GS_{i}}∥T_{1}\right)$. Then, the encrypted identity message $M_{1}=\left\{UID_{i},R_{i}⊕h\left(K_{GS_{i}}∥T_{1}\right),T_{1}\right\}$ is organized and sent to GWN.
Step 2 As receiving $M_{1}$, GWN verifies $T_{1}$ firstly. GWN calculates $h(K_{GS_{i}}∥T_{1})$ and obtains $R_{i}⊕h\left(K_{GS_{i}}∥T_{1}\right)$ by Exclusive-OR (XOR) operation. Then, $R_{i}$ is derived by $\left(R_{i}⊕h\left(K_{GS_{i}}∥T_{1}\right)\right)⊕h\left(K_{GS_{i}}∥T_{1}\right)$. According to semigroup property from Definition 3, we have $X_{i}=T_{b_{i}}\left(R_{i}\right)\;\mathrm{mod}\;p=T_{a_{i}b_{i}}\left(X\right)\;\mathrm{mod}\;p$ and $X_{i}^{\prime }=T_{b_{i}}\left(R_{i+1}\right)\;\mathrm{mod}\;p=T_{a_{i+1}b_{i}}\left(X\right)\;\mathrm{mod}\;p$. Then, we use $Y_{i}=X_{i-1}⊕h(K_{GS_{i}}∥R_{i}∥T_{2}∥0)$ and $Y_{i}^{\prime }=X_{i}^{\prime }⊕h(K_{GS_{i}}∥R_{i}∥T_{2}∥1)$ to generate the authentication information $Auth_{GS_{i}}=h(K_{GS_{i}}∥R_{i}∥Y_{i}∥Y_{i}^{\prime })$ for verifying GWN. Eventually, the message $M_{2}=\left\{S_{n},Y_{i},Y_{i}^{\prime },Auth_{GS_{i}},T_{2}\right\}$, where $S_{n}=\left\{U_{1},U_{2},\ldots ,U_{n}\right\}$, including meeting member list and GWN authentication information is sent to $U_{i}$.
Step 3 Any other member $U_{i},\forall i\neq j,$ receives $M_{2}$ and verifies $T_{2}$ and GWN by $Auth_{GS_{i}}$. Next, $X_{i-1}$ and $X_{i}^{\prime }$ are derived by $Y_{i}⊕h(K_{GS_{i}}∥R_{i}∥T_{2}∥0)$ and $Y_{i}^{\prime }⊕h(K_{GS_{i}}∥R_{i}∥T_{2}∥1),$ respectively. Thus, we can compute $V_{i}=T_{a_{i}}\left(X_{i-1}\right)\;\mathrm{mod}\;p=T_{a_{i-1}b_{i-1}a_{i}}\left(X\right)\;\mathrm{mod}\;p$, and $V_{i-1}=T_{a_{i}}\left(X_{i}^{\prime }\right)\;\mathrm{mod}\;p=T_{a_{i+1}b_{i}a_{i}}\left(X\right)\;\mathrm{mod}\;p$. Then, the factor of the session key can be derived $W_{i}=V_{i}/V_{i-1}=\left(T_{a_{i-1}b_{i-1}a_{i}}\left(X\right)\;\mathrm{mod}\;p\right)/\left(T_{a_{i+1}b_{i}a_{i}}\left(X\right)\;\mathrm{mod}\;p\right)$. Finally, the message $M_{3}=\left\{UID_{i},W_{i},T_{3}\right\}$ is broadcasted to all users.
Step 4 $U_{i}$ verifies $T_{3}$ after receiving $M_{3}$, and then derives the session key $sk_{i}$ by the following process:
  $$\begin{array}{ccc} sk_{i} & = & {\left(V_{i}\right)}^{n}\times {\left(W_{i+1}\right)}^{n-1}\times {\left(W_{i+2}\right)}^{n-2}\times \ldots \times \left(W_{i-1}\right)\\ & = & V_{1}\times V_{2}\times \ldots \times V_{n}. \end{array}$$
  The authentication information $Auth_{i1}=h(S_{n}∥sk_{i}∥UID_{i}∥T_{3})$ applied by other members, and the authentication information $Auth_{i2}=h(K_{GS_{i}}∥S_{n}∥V_{i}∥T_{3})$ applied by GWN can be derived. $U_{i}$ broadcasts authentication information $M_{4}=\left\{Auth_{i1},Auth_{i2}\right\}$ to other users.
Step 5 After receiving $M_{4}$, any other member $U_{j},\forall i\neq j,$ can authenticate $U_{i}$ by $Auth_{i1}$, and GWN can authenticate $U_{i}$ by $Auth_{i2}$. Eventually, the session key of this meeting can be generated $SK=h(S_{n},sk_{i})$.

When each participant obtains SK, they can start to communicate with each other via encrypting/decrypting messages by SK.

In PL-GAKA, we apply a chaotic map to reduce the computation cost from SGPAKE. The process of key agreement can be finished early, and the meeting members can build a safety communication. For the security, each participant applies a semigroup property shown in Definition 3 to compute the factor of session key as shown in Step 2. The messages required by the process of key agreement can be sent via the Internet. In summary, the proposed PL-GAKA requires low computation cost but provides similar security level to the Diffie–Hellman problem in a convenient cloud meeting.

## 4. Performance Analysis

We analyze the proposed solution in terms of the correctness, the security and the overall comparison with related solutions. For the security verification, we refer to [8,18,19,20,21,22] to evaluate session key security, mutual authentication, perfect forward security, and data integrity. Moreover, we also demonstrate that the proposed solution is safe when suffering replay attacks, impersonation attacks, privileged insider attacks, and stolen-verifier attacks.

### 4.1. Correctness

If each $U_{i}$ computes $sk_{i}$ correctly, it implies that all members have security communications in the cloud meeting. Therefore, we trace the process of generating $sk_{i}$, and the resuls are correct:

$$\begin{array}{ccc} sk_{i} & ={\left(T_{a_{i-1}b_{i-1}a_{i}}\left(x\right)\;\mathrm{mod}\;p\right)}^{n} & \times {\left(\frac{T_{a_{i}b_{i}a_{i+1}}\left(x\right)\;\mathrm{mod}\;p}{T_{a_{i-1}b_{i-1}a_{i}}\left(x\right)\;\mathrm{mod}\;p}\right)}^{n-1}\times {\left(\frac{T_{a_{i+1}b_{i+1}a_{i+2}}\left(x\right)\;\mathrm{mod}\;p}{T_{a_{i}b_{i}a_{i+1}}\left(x\right)\;\mathrm{mod}\;p}\right)}^{n-2}\\ & & \times \ldots \times (\frac{T_{a_{i+n-1}b_{i+n-1}a_{i+n}}\left(x\right)\;\mathrm{mod}\;p}{T_{a_{i+n-2}b_{i+n-2}a_{i+n-1}}\left(x\right)\;\mathrm{mod}\;p})\\ & =(T_{a_{i-1}b_{i-1}a_{i}}\left(x\right)\;\mathrm{mod}\;p) & \times \left(T_{a_{i}b_{i}a_{i+1}}\left(x\right)\;\mathrm{mod}\;p\right)\times \left(T_{a_{i+1}b_{i+1}a_{i+2}}\left(x\right)\;\mathrm{mod}\;p\right)\\ & & \times \ldots \times (T_{a_{i+n-1}b_{i+n-1}a_{i+n}}\left(x\right)\;\mathrm{mod}\;p)\\ & =(T_{a_{1}b_{1}a_{2}}\left(x\right)\;\mathrm{mod}\;p) & \times \left(T_{a_{2}b_{2}a_{3}}\left(x\right)\;\mathrm{mod}\;p\right)\times \left(T_{a_{3}b_{3}a_{4}}\left(x\right)\;\mathrm{mod}\;p\right)\\ & & \times \ldots \times (T_{a_{n}b_{n}a_{n+1}}\left(x\right)\;\mathrm{mod}\;p). \end{array}$$

### 4.2. Security Analysis

#### 4.2.1. Session Key Security

$U_{i}$ uses the session key to encrypt the information sending over Internet. Therefore, if the session key is secure, it means that the communication in the cloud meeting is also security. The proposed solution has the Diffie–Hellman problem. Even if attackers capture $T_{a_{i}}\left(x\right)$ or $T_{b_{i}}\left(x\right)$, they still can not generate authentication information. Moreover, we consider random value $a_{i}$ and $b_{i}$, so it is difficult for attackers to compute $sk_{i}$ and $SK=h(S_{n},sk_{i})$. Therefore, the session key is security in PL-GAKA.

#### 4.2.2. Mutual Authentication

In the authentication process, the authentication information is used to verify members and GWN. In PL-GAKA, each member uses $Auth_{GS_{i}}$ and $Auth_{i1}$ to verify GWN and other members while GWN uses $Auth_{i2}$ to verify participants. Even if attackers can capture the identity and $K_{GS_{i}}$, respectively, and then generate $Auth_{GS_{i}}$ and $M_{4}$, each member must be authenticated by all other members and GWN. Therefore, the PL-GAKA is secure under the multi-authentication consideration.

#### 4.2.3. Perfect Forward Security

Considering a situation in which attackers have the ability to capture the session key, they can use the session key to decrypt the information sending during cloud meetings. For example, a web user uses a username and a password to log in to a web service. If someone knows the username and the password, he/she can log in to the same web service and use it.

PL-GAKA does not take username and password into account for each meeting member. In each meeting, we use $sk_{i}={\left(V_{i}\right)}^{n}\times {\left(W_{i+1}\right)}^{n-1}\times {\left(W_{i+2}\right)}^{n-2}\times \ldots \times \left(W_{n-1}\right)$ to compute the session key $SK=h(S_{n},sk_{i})$. In other words, even if the session key is captured by attackers, the cloud meeting is still secure during the cloud meeting.

#### 4.2.4. Data Integrity

When the information is modified by attackers, we say that the protocol has data integrity if each member can recognize the correctness of the received data. In PL-GAKA, if $R_{i}⊕h\left(K_{GS_{i}}∥T_{1}\right)$ in $M_{1}$ is tampered with, GWN can use $h(K_{GS_{i}}∥T_{1})$ to capture $R_{i}$. If $W_{i}$ in $M_{3}$ is tampered with, other members will derive an unmatched $sk_{i}$. Therefore, the proposed protocol satisfies data integrity.

#### 4.2.5. Replay Attack

Attackers can eavesdrop on the packets sending over Internet to capture the communication information. Then, attackers send the captured information again to be an authenticated user. This is the replay attack. If the mechanism can not detect replay attack, someone can counterfeit an authentication member.

In the proposed solution, we consider the timestamp for each message. If attackers counterfeit an authentication member and resend the message again, the timestamp can be used to capture the irrationality. Thus, the replay attack is useless in PL-GAKA.

#### 4.2.6. Impersonation Attack

Impersonation attack means that illegal users impersonate legal ones and pass the authentication process with the stolen authenticated message to enter the system.

In the proposed group authenticated key agreement mechanism, the attacker can not obtain the authenticated message of $K_{GS_{i}}$ because $K_{GS_{i}}$ is encrypted. Without $K_{GS_{i}}$, the attacker can not impersonate $U_{i}$ or GWN. Therefore, PL-GAKA can defend impersonation attacks.

#### 4.2.7. Privileged-Insider Attack

Privileged-insider attack means that an authentication member impersonates other legal users with his/her own authenticated message. $U_{i}$ in PL-GAKA gets $K_{GS_{i}}$ from GWN in a safety tunnel in the registration process. Since different members will have various $K_{GS_{i}}$, no member can use his/her own $K_{GS_{i}}$ to impersonate the other one. Hence, this mechanism can defend privileged-insider attack.

#### 4.2.8. Stolen-Verifier Attack

Some protocol considers static verification data, which is saved in the server for authenticating members. Attackers steal the verification data from authentication servers, so that the attackers are authenticated by the verification data. Each member in the proposed solution is verified by other members and GWN, so verification data is not necessary. Therefore, the stolen-verifier attack is useless for the PL-GAKA.

#### 4.2.9. Shared Device

Sharing a communication device, e.g., cell phone or tablet, is a common behavior between friends. In our scenario, if the encryption and decryption protocols are implemented in the specific communication device, the sharing device may be a security issue. PL-GAKA requires users to provide the identity as shown in several processes, such as generating $M_{1}$ and $M_{3}$. If a sharing device is used in PL-GAKA, the impersonator still can not join the cloud meeting due to the lack of identity. Therefore, sharing a device does not work in PL-GAKA.

#### 4.2.10. Man-in-the-Middle Attack

During the key generation process, man-in-the-middle attack means that there is an attacker who builds a pair of connections with a specific sender and receiver. In other words, all messages sent from sender to receiver will be relayed by the attacker, and the attacker can access all the information of sender and receiver.

Man-in-the-middle attack is useless in the PL-GAKA, and we have the following properties to prove this claim. First, each member uses his/her unique $UID_{i}$ in the registration and authentication processes. Thus, generating $UID_{i}$ is an essential requirement. Second, each member must register in the GWN by the $UID_{i}$. The attacker has to be verified by GWN. Third, $S_{n}$ is considered in Step 2 of authentication process. In other words, each meeting member must be verified by each other. Putting the above together, PL-GAKA avoids a man-in-the-middle attack.

### 4.3. Security Analysis via BAN Logic

We apply Burrows-Abadi-Needham (BAN) logic to verify the security of PL-GAKA in a formal analysis. PL-GAKA consists of registration and authentication phrases. Since registration phrase can be processed in a safety tunnel, we focus on the analysis in terms of the authentication phrase.

**Figure 4:**
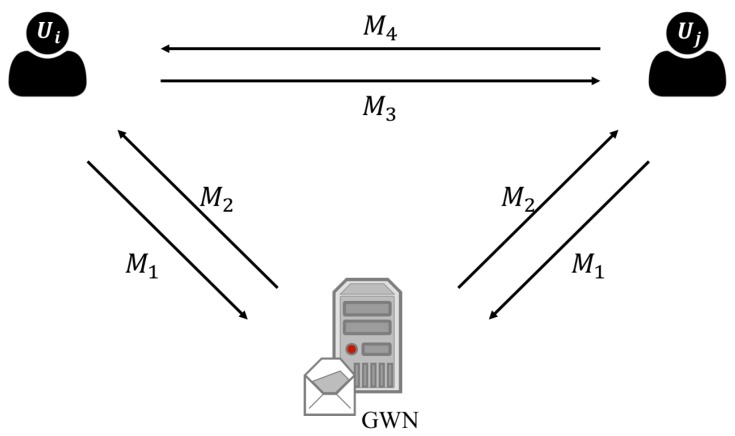
The message delivery structure in the authentication process of PL-GAKA.

PL-GAKA is a group key authentication scheme, and some cloud meeting members will exchange messages between each member and GWN. To simplify the communication model, we generalize a meeting communication to the model with GWN and two members $u_{i}$ and $u_{j}$. There are some concurrent processes in the authentication of PL-GAKA. For example, each member sends the identity message to GWN that all members send $M_{1}$ to GWN, and we consider a simple case that $u_{i}$ and $u_{j}$ send $M_{1}$ to GWN simultaneously. Moreover, $M_{3}$ and $M_{4}$ will be broadcasted to all members, and we consider the case that $u_{i}$ sends $M_{3}$ to $u_{j}$ while $u_{j}$ sends $M_{4}$ to $u_{i}$. Therefore, we can generalize the communication model to a simple one, as shown in Figure 4.

After registering in GWN, each $u_{i}$ has the initial state including $UID_{i}$, $K_{GS_{i}}$, and a timestamp generator. According to Figure 4, we have the following processes. Note that both $u_{i}$ and $u_{j}$ sends $M_{1}$ to GWN while GWN responses $M_{2}$ to $u_{i}$ and $u_{j}$, and we just focus on the notation on the communication between $u_{i}$ and GWN.

*P*_1_$said(u_{i},M_{1})$: $u_{i}$ sends $M_{1}$.*P*_2_$sees(GWN,M_{1})$: GWN receives $M_{1}$.*P*_3_$said(GWN,M_{2})$: GWN sends $M_{2}$.*P*_4_$sees(u_{i},M_{2})$: $u_{i}$ receives $M_{2}$.*P*_5_$said(u_{i},M_{3})$: $u_{i}$ sends $M_{3}$.*P*_6_$sees(u_{j},M_{3})$: $u_{j}$ receives $M_{3}$.*P*_7_$said(u_{j},M_{4})$: $u_{j}$ sends $M_{4}$.*P*_8_$sees(u_{i},M_{4})$: $u_{i}$ receives $M_{4}$.

Here, we have the following assumptions:
*A*_1_$bel(GWN,cont\left(u_{i},M_{1}\right))$: GWN believes that he/she has the ability to confirm $M_{1}$ sent from $u_{i}$.*A*_2_$bel(GWN,goodinfo\left(u_{i},M_{1},GWN\right))$: GWN believes that $M_{1}$ sent from $u_{i}$ to GWN is confirmed.*A*_3_$bel(u_{i},cont\left(GWN,M_{2}\right))$: $u_{i}$ believes that he/she has the ability to confirm $M_{2}$ sent from GWN.*A*_4_$bel(u_{i},goodinfo\left(GWN,M_{2},u_{i}\right))$: $u_{i}$ believes that $M_{2}$ sent from GWN to $u_{i}$ is confirmed.*A*_5_$bel(u_{j},cont\left(u_{i},M_{3}\right))$: $u_{j}$ believes that he/she has the ability to confirm $M_{3}$ sent from $u_{i}$.*A*_6_$bel(u_{j},goodinfo\left(u_{i},M_{3},u_{j}\right))$: $u_{j}$ believes that $M_{3}$ sent from $u_{i}$ to $u_{j}$ is confirmed.*A*_7_$bel(u_{i},cont\left(u_{j},M_{4}\right))$: $u_{i}$ believes that he/she has the ability to confirm $M_{4}$ sent from $u_{j}$.*A*_8_$bel(u_{i},goodinfo\left(u_{j},M_{4},u_{i}\right))$: $u_{i}$ believes that $M_{4}$ sent from $u_{j}$ to $u_{i}$ is confirmed.*A*_9_$bel(GWN,fresh\left(T_{1}\right))$: GWN believes that $T_{1}$ is fresh.*A*_10_$bel(u_{i},fresh\left(T_{2}\right))$: $u_{i}$ believes that $T_{2}$ is fresh.*A*_11_$bel(u_{j},fresh\left(T_{3}\right))$: $u_{j}$ believes that $T_{3}$ is fresh.

Thus, we have the following goals:
*G*_1_$bel(UID_{i},R_{i}⊕h\left(K_{GS_{i}}∥T_{1}\right),fresh\left(T_{1}\right))$. $GWN\to u_{i}$: $M_{1}$ sent from $u_{i}$ to GWN is correct and fresh.*G*_2_$bel(S_{n},Y_{i},Y_{i}^{\prime },Auth_{GS_{i}},fresh\left(T_{2}\right))$. $u_{i}\to GWN$: $M_{2}$ sent from GWN to $u_{i}$ is correct and fresh.*G*_3_$bel(UID_{i},W_{i},fresh\left(T_{3}\right))$. $u_{j}\to u_{i}$: $M_{3}$ sent from $u_{i}$ to $u_{j}$ is correct and fresh.*G*_4_$bel(Auth_{i1},Auth_{i2})$. $u_{i}\to u_{j}$: $M_{4}$ sent from $u_{j}$ to $u_{i}$ is correct and fresh.

From the believe connection, each goal can be achieved:
*G*_1_:From $P_{1}$, $P_{2}$, $A_{1}$, $A_{2}$, and $A_{9}$, $M_{1}$ is correct and fresh.*G*_2_:From $P_{3}$, $P_{4}$, $A_{3}$, $A_{4}$, and $A_{10}$, $M_{2}$ is correct and fresh.*G*_3_:From $P_{5}$, $P_{6}$, $A_{5}$, $A_{6}$, and $A_{11}$, $M_{3}$ is correct and fresh.*G*_4_:From $P_{7}$, $P_{8}$, $A_{7}$, and $A_{8}$, $M_{4}$ is correct and fresh.

Since each goal can be achieved, PL-GAKA provides a secure session key generation.

### 4.4. Security Comparison

The overall comparison between PL-GAKA and related approaches are shown in Table 2. We refer to [8] for considering the following protocols:

- Protocol #1 proposed by Abdalla and Pointcheval is a group password-based key agreement [15].
- Protocol #2 proposed by Dutta and Barua is a group password-based authentication key agreement [16].
- Protocol #3 proposed by Kim et al. is a group key agreement [3].
- Protocol #4 proposed by Boyd and Nieto is a group key agreement [17].
- Protocol #5 proposed by Lee et al. is a group password-based authentication key agreement [8].

For the security consideration, PL-GAKA takes into account the extended chaotic map to improve the computation efficiency from SGPAKE. Although the extended chaotic map does not provide the Diffie–Hellman problem, we still can derive an equivalent security level by extended Chebyshev polynomials. Therefore, solving the message generated by the extended chaotic map requires similar computing resource to that in the Diffie–Hellman problem. Therefore, the security level gap between PL-GAKA and SGPAKE is small.

### 4.5. Efficiency Comparison

The results of the efficiency comparison between SGPAKE and PL-GAKA are illustrated in Table 3. Since this paper focuses on the cloud meeting and improves SGPAKE in the cloud meeting, we compare PL-GAKA with SGPAKE. For the Exponentiation evaluation, SGPAKE requires $4(2^{a})$ because of two modular exponential computations for generating session keys. According to the properties of cloud meetings, the participant list can be determined before PL-GAKA starts, so the heavy work can be well prepared, and the computation cost can be finished from an offline computation.

For the efficiency of the session key calculation process, PL-GAKA considers the extended chaotic map, which is a lightweight calculation compared with the modular exponential computation. Thus, PL-GAKA requires less computation time to generate a session key than that of SGPAKE. On the other hand, the meeting member does not require a password to verify the identity in PL-GAKA, so the password maintenance mechanism is not necessary in Pl-GAKA, but it is required in SGPAKE. Putting the above together, PL-GAKA is more efficient than SGPAKE in terms of key generation and the user maintenance.

## 5. Conclusions

Group authentication key agreement is necessary for providing security communications, and a cloud meeting is a typical and popular application. Lee et al. present SGPAKE to realize the secure group communication. However, SGPAKE is a Diffie–Hellman key exchange scheme, and the heavy computation cost is an implementation issue. We consider SGPAKE and apply the extended chaotic map to propose a password-less group authentication key agreement named PL-GAKA. Since an extended chaotic map provides properties that are similar to semigroup in chaotic map, the security of PL-GAKA is equivalent to that of SGPAKE. PL-GAKA is a password-less protocol, so each user does not worry about the password maintenance. Moreover, the session key is dynamic in each cloud meeting. In other words, PL-GAKA considers short-term authentication, and it provides stronger security than other long-term authentication protocols. In the future, we will focus on the progress on improving the registration security of the meeting members coming from various companies, and consider sharing devices.

When a cloud meeting takes place, only the registered users can be invited to join the meeting. In the real world applications, the registration can be finished when a new staff member is reported to the company, and the entire process can be done in a secure procedure. It means that the meeting members must be employed in the same company in PL-GAKA. In other words, the registration process must be improved for staff members from different companies that do not have consistent registration processes.

## Figures and Tables

**Table 1:** The symbol system applied by the proposed solution.

Symbol	Definition
$U_{i}$	*i*-th user
GWN	The trusty authentication server
h(.)	One-way hash function
$K_{G}$	The private key generated by GWN
$UID_{i}$	The id of $U_{i}$
*p*	A large prime number
$T_{r}$	Chaotic map
*T*	The timestamp
*x*	A variable within (−∞,∞)
$K_{GS_{i}}$	The identity of GWN for $U_{i}$
$Auth_{GS_{i}}$	The authentication information applied by $U_{i}$ for verifying GWN
$Auth_{i1}$	The authentication information applied by $U_{j}$, ∀j≠i, for verifying $U_{i}$
$Auth_{i2}$	The authentication information applied by GWN for verifying $U_{i}$
$sk_{i}$	The factor of generating session key for $U_{i}$
$S_{n}$	The list of participants
SK	The session key

**Table 2:** The overall comparison between the proposed solution and related approaches.

Protocol	Protocol #1	Protocol #2	Protocol #3	Protocol #4	Protocol #5	PL-GAKA
Public Key	No	No	Yes	Yes	No	No
Private Key	shared password	shared password	PKI-based	PKI-based	Yes	No
Asymmetric Encryption	No	No	No	Yes	No	No
Symmetric Encryption	Yes	Yes	No	No	Yes	No
Signature Verification	No	No	Yes	Yes	No	No
Mutual Authentication	No	Yes	No	No	Yes	Yes
Perfect Forward Security	Yes	No	Yes	No	Yes	Yes

PKI: Public Key Infrastructure.

**Table 3:** The efficiency comparison between SGPAKE and PL-GAKA.

Protocol	SGPAKE	PL-GAKA
Password Maintenance	Yes	No
Exponentiation	Yes	No
Key Calculation	Modular Exponentiation	Extented Choatic Map

## Abbreviations

The following abbreviations are used in this manuscript:

GWN	Trust Authentication Server
SGPAKE	Simple Group Password-based Authenticated Key Agreement
PKI	Public Key Infrastructure
PL-GAKA	Passwordless Group Authentication Key Agreement
